# Causal roles of prefrontal and temporo-parietal theta oscillations for inequity aversion

**DOI:** 10.1093/scan/nsad061

**Published:** 2023-11-01

**Authors:** Patricia Christian, Georgia E Kapetaniou, Alexander Soutschek

**Affiliations:** Department of Psychology, Ludwig Maximilians University Munich, Munich, Germany; Graduate School of Systemic Neurosciences, Department of Biology, Ludwig Maximilians University Munich, Munich, Germany; Department of Psychology, Ludwig Maximilians University Munich, Munich, Germany; Graduate School of Systemic Neurosciences, Department of Biology, Ludwig Maximilians University Munich, Munich, Germany; Department of Psychology, Ludwig Maximilians University Munich, Munich, Germany; Graduate School of Systemic Neurosciences, Department of Biology, Ludwig Maximilians University Munich, Munich, Germany

**Keywords:** transcranial alternating current stimulation (tACS), social decision making, perspective taking, temporo-parietal junction, lateral prefrontal cortex, equity

## Abstract

The right temporo-parietal junction (rTPJ) and the right lateral prefrontal cortex (rLPFC) are known to play prominent roles in human social behaviour. However, it remains unknown which brain rhythms in these regions contribute to trading-off fairness norms against selfish interests as well as whether the influence of these oscillations depends on whether fairness violations are advantageous or disadvantageous for a decision maker. To answer these questions, we used non-invasive transcranial alternating current stimulation (tACS) to determine which brain rhythms in rTPJ and rLPFC are causally involved in moderating aversion to advantageous and disadvantageous inequity. Our results show that theta oscillations in rTPJ strengthen the aversion to unequal splits, which is statistically mediated by the rTPJ’s role for perspective taking. In contrast, theta tACS over rLPFC enhanced the preference for outcome-maximizing unequal choices more strongly for disadvantageous compared to advantageous outcome distributions. Taken together, we provide evidence that neural oscillations in rTPJ and rLPFC have distinct causal roles in implementing inequity aversion, which can be explained by their involvement in distinct psychological processes.

## Introduction

Fairness motives play an important role in guiding human social behaviour by determining which payoff allocations are considered as desirable. Previous findings suggest that humans are averse to inequity both when they receive lower (disadvantageous inequity) and higher payoffs than others (advantageous inequity) (Fehr and Schmidt, 1999). Aversion to advantageous and disadvantageous inequity are hypothesized to relate to distinct psychological processes: While advantageous inequity aversion is discussed to rely on mentalizing processes enabling humans to take the perspective of others (Underwood and Moore, 1982; Imuta *et al.*, 2016), overcoming aversion to disadvantageous inequity may require downregulating the negative emotional reactions to unfair allocations (McAuliffe *et al.*, 2017). Despite the evidence that distinct psychological motives underlie prosocial behaviour in the domains of advantageous and disadvantageous inequity, less is known about whether social decision making in these domains is implemented by dissociable brain mechanisms. While previous research ascribes the right temporo-parietal junction (rTPJ) and the right lateral prefrontal cortex (rLPFC) central roles in trading-off selfish interests against fairness norms (Strang *et al.*, 2015; Strombach *et al.*, 2015; Will *et al.*, 2015; Yamagishi *et al.*, 2016; Maier *et al.*, 2018; Cutler and Campbell-Meiklejohn, 2019; Speer and Boksem, 2019), the precise roles of these regions for advantageous or disadvantageous inequity aversion are poorly understood.

The rTPJ is thought to promote prosociality toward others, but there is disagreement on whether the rTPJ generally encodes reward values for others (Hare *et al.*, 2010; Hutcherson *et al.*, 2015; Park *et al.*, 2017) or whether the rTPJ is more specifically involved in resolving conflicts between self- and other-regarding motives under advantageous inequity (Morishima *et al.*, 2012; Soutschek *et al.*, 2016; Obeso *et al.*, 2018). The rTPJ’s function for fairness-guided behaviour is often explained by its more general role for perspective taking (Morishima *et al.*, 2012; Strombach *et al.*, 2015; Soutschek *et al.*, 2016). Previous electrophysiological findings on brain rhythms underlying higher-level cognitive functioning suggest that perspective taking is associated with theta oscillations in the rTPJ (Wang *et al.*, 2016; Gooding-Williams *et al.*, 2017; Seymour *et al.*, 2018). Consistent with this, previous research linked prosocial choices to temporo-parietal theta oscillations (Billeke *et al.*, 2014), though other studies reported correlations between prosociality and beta, rather than theta, oscillations in the rTPJ (Gianotti *et al.*, 2019). These inconsistent findings raise the question as to whether perspective taking and social decision making are implemented by the same or dissociable brain rhythms within the rTPJ.

Likewise, also the role of the rLPFC for prosocial choices is controversially debated. The rLPFC has been hypothesized to play a key role in resolving conflicts between selfish interests and fairness considerations when being confronted with unfairness (Buckholtz *et al.*, 2008, 2015). Previous finding suggest that rLPFC promotes the rejection of unfair offers even if this reduces one’s own payoff (Knoch *et al.*, 2006; Baumgartner *et al.*, 2011), which is consistent with a more general role of the rLPFC for goal-directed actions and cognitive control (Miller and Cohen, 2001; Mansouri *et al.*, 2009; Yamagata *et al.*, 2012; Muhle-Karbe *et al.*, 2018). However, imaging studies directly comparing rLPFC activation between advantageous and disadvantageous inequity inconsistently reported stronger rLPFC activation either during advantageous inequity (Gao *et al.*, 2018) or, in contrast, during disadvantageous inequity (Fliessbach *et al.*, 2012). Thus, the rLPFC’s role for moderating aversion to disadvantageous or advantageous inequity is far from understood. Further, even though previous research suggests that control processes in the rLPFC during negative feedback and conflict processing are associated with theta oscillations (van de Vijver *et al.*, 2011; Oehrn *et al.*, 2014), the specific brain rhythms underlying the rLPFC’s role for conflicts between fairness-guided behaviour and selfish interests remain unknown.

To resolve the controversy between conflicting accounts on rTPJ and rLPFC functioning in social decision making, we conducted two experiments assessing the causal roles of rTPJ and rLPFC oscillations for social decision making with transcranial alternating current stimulation (tACS). tACS is a non-invasive brain stimulation method that allows modulating brain rhythms in a frequency-specific manner, providing insights into the causal relationships between specific brain oscillations and (social) behaviour (Polanía *et al.*, 2018). In particular, we tested the following hypotheses: First, given the crucial role of theta oscillations for perspective taking in the rTPJ (Wang *et al.*, 2016; Gooding-Williams *et al.*, 2017; Seymour *et al.*, 2018), we hypothesized that theta tACS over rTPJ strengthens inequity aversion by increasing the sensitivity to conflicts between selfish and other-regarding interests. More specifically, we expected rTPJ tACS to increase advantageous inequity (Morishima *et al.*, 2012; Soutschek *et al.*, 2016; Obeso *et al.*, 2018), but rTPJ stimulation may additionally affect also aversion to disadvantageous inequity if the rTPJ plays a general role for resolving conflicts between selfish and other-regarding interests. Second, we expected that entrainment of theta oscillations in rLPFC reduces aversion to disadvantageous rather than advantageous outcomes due to the involvement of prefrontal theta in cognitive control (van de Vijver *et al.*, 2011; Oehrn *et al.*, 2014).

## Materials and methods

### Participants

We tested 64 volunteers who were recruited at the Ludwig Maximilians University. We excluded data from three participants due to technical issues with tACS and from one participant due to electrode movement during the experiment, leaving 30 participants for the rTPJ experiment (17 female, *M*_age_ = 25.2 years, *SD*_age_ = 3.8 years) and 30 participants for the rLPFC experiment (14 female, *M*_age_ = 23.4 years, *SD*_age_ = 4.2 years). According to a power analysis assuming the effect size of Cohen’s *d* = 0.54 observed in a previous tACS study on decision making (Soutschek *et al.*, 2021), 29 participants are sufficient to detect significant effects (*P* = 0.05, two-tailed) with a power of 80% (though we note that this power analysis may underestimate the required sample size for detecting higher-order interaction effects, as studied in the current experiment). All participants were healthy volunteers, without any known psychiatric or neurological disorders or contra-indications for tACS. The study was approved by the local ethics committee and conducted following the principles of the Declaration of Helsinki (2013) as well as the safety guidelines for tACS (Bikson *et al.*, 2016). All participants gave informed written consent prior to participation and received a payment of 10 Euro/h as well as additional earnings from the social decision task (dictator game).

### Dictator game

**Fig. 1.**
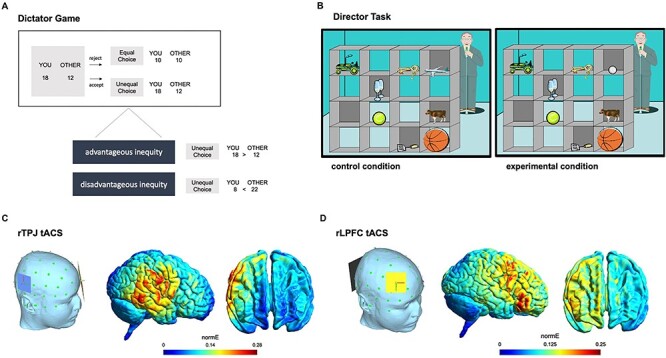
(A) Example trial of the dictator game. Participants had to decide whether to accept or reject a proposed unequal payoff for themselves and another player within 4 s. The displayed payoffs (in coins) could either be advantageous (*M*_self_ > *M*_other_) or disadvantageous (*M*_other_ > *M*_self_) for the participant. If participants rejected the unequal split, both the participant and the other player received a fixed amount of 10 coins (equal choice option). (B) Example trial of the director task: participants had to follow the auditory instructions of the director and select the designated target object visible from the director’s view (‘Where is the small ball?’). In control trials, two objects belonging to the same category were presented (e.g. two balls), but only one of the objects matched the exact instruction of the director and was visible from both perspectives (in this example, the small yellow ball). In experimental trials, the director’s view was incongruent with the participant’s one (here, the smallest, white ball is occluded from director’s view). To identify the target object (in the example, the yellow ball, as it is the smallest ball visible from director’s perspective), participants had to inhibit their own perspective and take the director’s perspective instead. (C/D) Simulations of electric current flow with the SimNIBS toolbox (Saturnino *et al.*, 2019) for the (C) rTPJ and (D) rLPFC electrode placement.

Participants played an adapted version of the dictator game (Hutcherson *et al.*, 2015; Kapetaniou *et al.*, 2021) implemented in Matlab 2019a (Mathworks, Inc., Sherborn, MA) using the Cogent toolbox. Participants played in the role of the proposer (‘dictator’) and decided how to split a sum of coins between themselves (*M*_self_) and another anonymous player (*M*_other_). On each trial, an unequal choice option with different payoffs for the dictator and the other was displayed on the screen and participants had to decide within 4 s whether to accept or reject the unequal split. The monetary split could either be advantageous (proposer obtains higher payoff than receiver, e.g. ‘18 coins for you, 12 for the other’) or disadvantageous (proposer obtains less than receiver, e.g. ‘6 coins for you, 12 for other’) for the participant (Figure 1A). If participants rejected the unequal choice option, both the participant and the other player received a fixed amount of 10 coins (equal choice option). If participants failed to respond within 4 s, both players gained 0 coins. For the unequal choice option, the amounts for *M*_self_ and *M*_other_ varied from 1 to 31 coins (see Supplementary material), allowing us to disentangle efficiency concerns (combined payoff for both participants: *M*_self_ + *M*_other_) and absolute inequity (|*M*_self_ − M_other_|) (Gao *et al.*, 2018; Kapetaniou *et al.*, 2021). The dictator game included a total of 96 trials with equal numbers of advantageous and disadvantageous choice options. We also included catch trials where the unequal option was replaced by another equal option involving either higher (e.g. ‘18 coins for you, 18 for other’) or lower stakes than the standard equal option (10 coins for both) to test participants’ task understanding. Choice options were presented in random order to avoid repetition effects. Participants indicated their choices to accept or reject the unequal split via pressing the left or right control keys on a standard keyboard (key-choice assignment was counterbalanced across participants). We informed participants that their choices had real consequences for themselves and the other: At the end of the experiment, one choice was randomly selected and the participant (*M*_self_) and as well as the next participant coming to the lab (*M*_other_) received a monetary bonus based on the participant’s decision (with an exchange rate of 5 coins = 1 euro).

### Director task

We used an adapted version of the director task to measure perspective taking (Dumontheil *et al.*, 2010; Santiesteban *et al.*, 2012, 2015; Symeonidou *et al.*, 2016; Tamnes *et al.*, 2018). In this task, a 4 × 4 set of shelves containing eight different objects and a human agent (‘director’) standing behind the shelves were displayed on the screen. All objects were visible from the participant’s perspective, whereas some objects were occluded from the director’s perspective (Figure 1B). Some of the presented objects belonged to the same category (e.g. balls), but differed in position (upper vs. lower shelves) or size (large vs. small). Participants had to follow the auditory instructions of the director (e.g. ‘Where is the small ball?’; instructions were given in German) and decide which target object was visible from the director’s perspective. For example, if the director asked for the location of the small ball, participants had to decide whether the smallest ball visible for the director was located on the left or right side of the shelf. Auditory instructions were presented for 2.2 s and started simultaneously with the display of the visual stimulus. Then, participants had to indicate whether the target stimulus was positioned on the right or the left side within 3.6 s by pressing the right or left key (target locations were counterbalanced across trials), in accordance with past research measuring perspective taking with the director task (Dumontheil *et al.*, 2010; Santiesteban *et al.*, 2012, 2015). In control trials, the target object was visible from both the participant’s and the director’s perspective (congruent perspectives), such that participants could stick with their own perspective to select the target object. In contrast, in experimental trials the object which fitted to the auditory instructions from the participant’s perspective (e.g. the smallest ball in the shelf) was occluded from the director’s view (incongruent perspectives). To resolve the conflict between the incongruent perspectives, participants had to inhibit their own perspective and select the target object visible from the director’s position (Dumontheil *et al.*, 2010). Stimuli were presented in counterbalanced order across participants. To avoid repetition effects, no stimulus was presented more than once. The director task included a total of 96 trials (48 experimental and 48 control trials in random order). In line with previous research (Dumontheil *et al.*, 2010; Santiesteban *et al.*, 2012, 2015), the director task included no catch trials, contrary to the dictator game.

### tACS protocol

We used a 16-channel tDCS stimulator (neuroConn, Ilmenau, Germany) to apply tACS with sham, theta (6 Hz), or beta (20 Hz) stimulation frequency. For rTPJ stimulation, a smaller (5 × 5 cm) saline-soaked sponge electrode was placed vertically over electrode position CP6 (Santiesteban *et al.*, 2012) and a larger (10 × 10 cm) electrode was placed horizontally over electrode position FP1 according to the 10/20 system. For rLPFC stimulation, the smaller electrode (5 × 5 cm) was placed horizontally over electrode position F4 and the larger electrode (10 × 10 cm) was placed horizontally over the occipital lobe (Frings *et al.*, 2018) (Figure 1D). We applied online tACS during task performance with a current strength of 1 mA (peak-to-peak). Following previous procedures (Moisa *et al.*, 2016; Soutschek *et al.*, 2021, 2022), tACS was administered during task performance in miniblocks lasting <3 min in order to minimize the risk of tACS-induced aftereffects. Each stimulation block started with a ramp-up phase for the tACS current for 15 s, followed by a buffer interval of 15 s before the start of the task to allow stimulation effects on brain activity to build up before task performance (Nitsche and Paulus, 2001; Vosskuhl *et al.*, 2016). In the sham condition, the current was ramped down directly after the ramp-up phase. During task performance, participants received online stimulation either for 152 s (dictator game) or for 122 s (director task). After each miniblock, participants had to indicate the perceived aversiveness of the stimulation on a rating scale from 0 (not aversive at all) to 20 (very aversive) within 5 s as measure of tACS-induced discomfort. The following block started after a stimulation-free interval of 35 s (including the ramp down phase of 5 s) to minimize carry-over effects between tACS conditions. In both experiments, the order of stimulation conditions was counterbalanced using Latin square methods.

### Experimental design

Experimental procedures were identical for the rTPJ and rLPFC experiments (apart from the electrode placement). Both experiments followed a within-subject design in which participants performed two experimental tasks (dictator game and director task), while undergoing sham, theta, or beta tACS. In line with safety regulations for tACS, participants were screened for possible tACS exclusion criteria and informed about possible side effects (Bikson *et al.*, 2016). Participants received detailed task instructions and performed a short practice block for each task (without tACS). During tACS, participants performed 6 miniblocks of the dictator game (22 trials each) and 6 miniblocks (16 trials each) of the director task. The task order was counterbalanced across participants. At the end of the experiment, participants filled in a questionnaire asking for demographic data and the perceived aversiveness of tACS on a rating scale from 0 (not aversive at all) to 10 (very aversive). In total, one session lasted ∼1 h 30 min.

### Statistical analysis

We analysed data of the dictator game with Bayesian generalized linear mixed models (GLMMs) as implemented in the brms package in R (Bürkner, 2017). Following the procedures, we had established in our previous study (Kapetaniou *et al.*, 2021), we therefore assessed the impact of rTPJ and rLPFC tACS on social decision making as a function of the degree of inequity between the participant’s and the receiver’s payoff (Inequity_absolute_ = |*M*_self_ − *M*_other_|) as well as the efficiency of an offer, i.e. the overall payoff for both participants (Efficiency = *M*_self_ + *M*_other_). In more detail, for both the rTPJ and the rLPFC experiment, we performed Bayesian GLMMs regressing binary choices (0 = equal option, 1 = unequal option) on fixed-effect predictors for tACS_theta-sham_, tACS_beta-sham_, Inequity_type_ (0 = advantageous inequity, 1 = disadvantageous inequity), Inequity_absolute_, Efficiency and the interaction terms. We also included discomfort ratings after each tACS block as predictors of no interest to control for potential confounding effects of tACS-induced discomfort on choices. All fixed-effect predictors were also modelled as random slopes in addition to participant-specific random intercepts. Continuous predictors were *z*-transformed. We assessed the statistical significance of model parameters with the 95% highest density interval (HDI) of the posterior distributions (Kruschke, 2010; Ahn *et al.*, 2017). Parameter values falling within the 95% HDI are considered as more credible than parameter values outside of the HDI (Kruschke, 2013, 2018). If the 95% HDI does not overlap with zero, parameter estimates are considered as statistically significant (Kruschke, 2013), in analogy to frequentist statistics. To minimize the impact of priors on the parameter estimates, we used weakly informative flat uniform distributions as priors as implemented in the brms package (Bürkner, 2017). The model was fitted with two Markov chain Monte Carlo (MCMC) chains with 3000 iterations, including 1000 warm-up iterations. We used $\hat R$ as measure of model convergence: $\hat R$ was below 1.01 for all parameter estimates, suggesting model convergence.

Additionally, we analysed choices in the dictator game with computational models of inequity aversion. We first considered a simple model where the utility of a decision maker’s payoff is reduced with increasing inequity between her own and the other person’s payoff (weighted with a participant-specific parameter *κ* that captures an individual’s inequity aversion):


(1) $${\mathrm{U = }}{{\mathrm{M}}_{{\mathrm{self}}}}{{- \kappa \times | }}{{\mathrm{M}}_{{\mathrm{self}}}}{\mathrm{- }}{{\mathrm{M}}_{{\mathrm{other}}}}$$

More sophisticated models of inequity aversion (Fehr and Schmidt, 1999) assume that decision makers assign different weights to advantageous (*β*) and disadvantageous outcomes (*α*):


(2) $${\mathrm{U = }}{{\mathrm{M}}_{{\mathrm{self}}}}{{- \beta \times max}}\left( {{{\mathrm{M}}_{{\mathrm{self}}}}{\mathrm{- }}{{\mathrm{M}}_{{\mathrm{other}}}}{\mathrm{, 0}}} \right){{ - \alpha \times max}}\left( {{{\mathrm{M}}_{{\mathrm{other}}}}{\mathrm{- }}{{\mathrm{M}}_{{\mathrm{self}}}}{\mathrm{, 0}}} \right)$$

We note that the Fehr–Schmidt model is equivalent to the Charness–Rabin model for two-person games without reciprocity (Charness and Rabin, 2002). For both models, we translated utilities into probabilities of choosing the equal or unequal reward option with a standard softmax link function:


(3) $${\mathrm{P}}\left( {{\mathrm{choice\ of\ unequal\ option}}} \right) \nonumber\\ = {\left( {{\mathrm{1 + exp}}\left( {{\mathrm{-}}{{{\beta }}_{{\mathrm{temp}}}}{\mathrm{ \times }}\left( {{{\mathrm{U}}_{{\mathrm{unequal}}}}{\mathrm{- }}{{\mathrm{U}}_{{\mathrm{equal}}}}} \right)} \right)} \right)^{{\mathrm{ - 1}}}}$$

We estimated model parameters using the function fmincon in Matlab (2000 iterations), separately for each participant and tACS condition. We used 100 different starting values and extracted parameters for the best-fitting converging estimation for each participant (indicated by a low Akaike information criterion (AIC)). Parameter estimates were compared between tACS conditions with non-parametric Wilcoxon tests.

In the director task, we used Bayesian GLMMs to analyse performance accuracy. Accurate responses in experimental trials reflect a participant’s ability to take the perspective of the director in case of conflict between one’s own and the director’s incongruent perspectives, whereas in control trials, the participants’ and the director’s perspectives were congruent. We regressed binary responses (1 = correct, 0 = incorrect response) on fixed-effect predictors for tACS_theta-sham_, tACS_beta-sham_, Condition (1 = experimental, 0 = control) and the interaction terms. Again, we entered discomfort ratings as covariate of no interest. All fixed effects were modelled also as random slopes in addition to participant-specific intercepts. For the analysis of the director task, we used the same model fitting procedures as for the dictator game. $\hat R$ values were below 1.01 for all parameter estimates, suggesting that all models converged.

## Results

### Theta tACS over rTPJ and rLPFC affect dissociable aspects of inequity aversion

First, we tested the causal involvement of the rTPJ and rLPFC in advantageous and disadvantageous inequity aversion. For both the rTPJ and the rLPFC experiment, we regressed binary choices on predictors for tACS_theta-sham_, tACS_beta-sham_, Inequity_type_ (advantageous inequity = 0, disadvantageous inequity = 1), Inequity_absolute_, Efficiency and the interaction terms, controlling for tACS-induced discomfort.

In the rTPJ experiment, sanity checks revealed that under sham participants accepted unequal splits less often if inequity was disadvantageous compared to advantageous for them (for mean levels of Inequity_absolute_ and Efficiency), Inequity_type_: HDI_mean_ = −2.65, HDI_95%_ = [−4.49, −0.87], indicating a stronger aversion against disadvantageous than advantageous inequity (Fehr and Schmidt, 1999). They also preferred more efficient choices (for mean levels of Inequity_absolute_ under advantageous inequity), Efficiency: HDI_mean_ = 4.14, HDI_95%_ = [2.71, 5.77], as well as options with smaller absolute differences between *M*_self_ and *M*_other_ (for mean levels of Efficiency under advantageous inequity), Inequity_absolute_: HDI_mean_ = −1.32, HDI_95%_ = [−2.08, −0.58], the latter suggesting that participants were averse to advantageous inequity. Furthermore, participants were more averse to increasing disadvantageous compared to advantageous inequity (for mean levels of Efficiency), Inequity_absolute_ × Inequity_type_: HDI_mean_ = −2.15, HDI_95%_ = [−3.34, −1.05] and showed a stronger preference for efficient (i.e. payoff-maximizing) outcomes under disadvantageous in contrast to advantageous inequity (for mean levels of Inequity_absolute_), Efficiency × Inequity_type_: HDI_mean_ = 4.13, HDI_95%_ = [2.25, 6.42]. Taken together, participants’ preferences for unequal splits strongly depended on whether inequity was advantageous or disadvantageous for them.

**Fig. 2.**
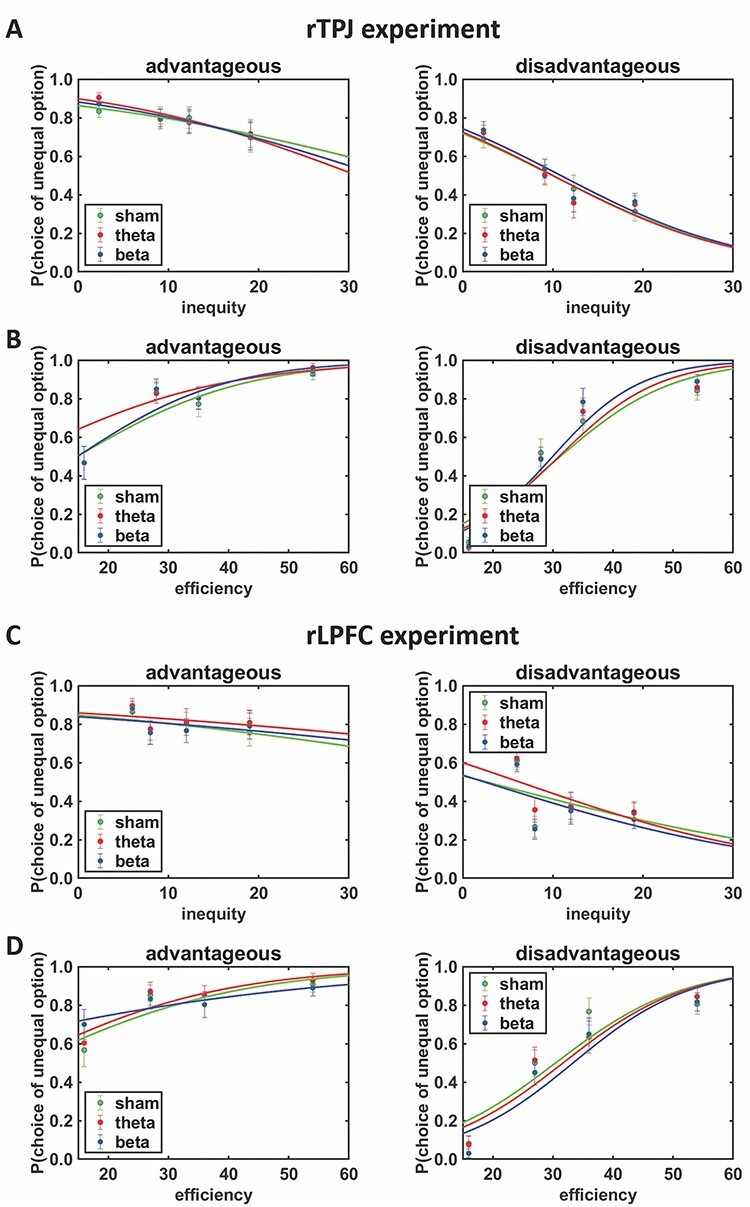
Stimulation effects on inequity aversion based on the results of Bayesian generalized linear mixed models in the dictator game. (A) Theta tACS over rTPJ increased aversion to advantageous inequity, but this effect was not significantly stronger than on disadvantageous inequity aversion. (B) We observed no rTPJ stimulation effects on efficiency concerns. In contrast, theta tACS over rLPFC did not affect (C) inequity aversion per se but increased (D) the preference for efficient options (i.e. unequal choice option that maximize the overall payoff for both players) more strongly under disadvantageous compared with advantageous inequity. Coloured dots represent binned mean raw data and error bars indicate SEM. Logistic curves show sigmoidal functions fitted to the binned raw data.

Next, we assessed how rTPJ tACS affected choices in the dictator game. We observed that tACS_theta-sham_ significantly increased advantageous inequity aversion (for mean levels of Efficiency; note that advantageous inequity was defined as reference category), tACS_theta-sham_ × Inequity_absolute_: HDI_mean_ = -0.73, HDI_95%_ = [−1.48, −0.04] (Table 1), but stimulation effects did not significantly differ between advantageous and disadvantageous inequity, tACS_theta-sham_ × Inequity_type_ × Inequity_absolute_: HDI_mean_ = 0.58, HDI_95%_ = [−0.47, 1.65] (Figure 2A–B). We observed no significant effects of tACS_beta-sham_ on Inequity_absolute_, Efficiency, or Inequity_type_ (Table 1), and also a further GLMM directly comparing theta and beta tACS revealed no significant stimulation effects. Thus, entrainment of theta oscillations in rTPJ increases aversion to unequal splits, with no significant differences between stimulation effects on advantageous and disadvantageous inequity aversion.

**Table 1.** Results of Bayesian GLMM for the dictator game in the rTPJ experiment. We report the upper and lower borders of the 95% HDI of the posterior distributions. SEM are in brackets. Significant effects are highlighted in bold

Predictor	Estimate (SE)	2.5%	97.5%
**Intercept**	**5.25 (0.95)**	**3.53**	**7.23**
tACS_theta-sham_	0.67 (0.43)	−0.13	1.57
tACS_beta-sham_	0.78 (0.44)	−0.07	1.65
**Inequity_absolute_**	**−1.32 (0.38)**	**−2.08**	**−0.58**
**Efficiency**	**4.14 (0.78)**	**2.71**	**5.77**
**Inequity_type_**	**−2.65 (0.89)**	−**4.49**	−**0.87**
Discomfort	−0.22 (0.21)	−0.66	0.18
**Inequity_absolute_ × Inequity_type_**	**−2.15 (0.58)**	−**3.34**	−**1.05**
Inequity_absolute_ × Efficiency	−0.31 (0.37)	−1.07	0.37
**Efficiency × Inequity_type_**	**4.13 (1.09)**	**2.25**	**6.42**
tACS_theta-sham_ × Inequity_type_	−0.45 (0.54)	−1.55	0.58
tACS_beta-sham_ × Inequity_type_	0.01 (0.56)	−1.12	1.09
**tACS_theta-sham_ × Inequity_absolute_**	**−0.73 (0.38)**	−**1.48**	−**0.04**
tACS_beta-sham_ × Inequity_absolute_	−0.50 (0.35)	−1.18	0.16
tACS_theta-sham_ × Efficiency	0.70 (0.51)	−0.27	1.77
tACS_beta-sham_ × Efficiency	0.73 (0.51)	−0.25	1.71
tACS_theta-sham_ × Inequity_absolute_ × Inequity_type_	0.58 (0.54)	−0.47	1.65
tACS_beta-sham_ × Inequity_absolute_ × Inequity_type_	0.43 (0.54)	−0.61	1.52
tACS_theta-sham_ × Efficiency × Inequity_type_	0.02 (0.76)	−1.47	1.47
tACS_beta-sham_ × Efficiency × Inequity_type_	0.46 (0.80)	−1.11	2.02
tACS_theta-sham_ × Inequity_absolute_ × Efficiency	−0.27 (0.51)	−1.28	0.71
tACS_beta-sham_ × Inequity_absolute_ × Efficiency	−0.62 (0.53)	−1.69	0.40
tACS_theta-sham_ × Inequity_absolute_ × Efficiency × Inequity_type_	−0.32 (0.75)	−1.77	1.16
tACS_beta-sham_ × Inequity_absolute_ × Efficiency × Inequity_type_	0.35 (0.80)	−1.30	1.89

A different pattern of stimulation effects emerged in the rLPFC experiment: In the sham condition, we again observed significant effects of Inequity_type_: HDI_mean_ = −4.56, HDI_95%_ = [−7.36, −1.99], Inequity_absolute_: HDI_mean_ = −1.57, HDI_95%_ = [−2.46, −0.64] and Efficiency: HDI_mean_ = 4.06, HDI_95%_ = [2.53, 5.97]. As in the rTPJ experiment, participants also showed stronger preferences for more efficient (for mean levels of Inequity_absolute_), Efficiency × Inequity_type_: HDI_mean_ = 4.59, HDI_95%_ = [2.34, 7.35] and less unequal options (for mean levels of Efficiency), Inequity_absolute_ × Inequity_type_: HDI_mean_ = -2.21, HDI_95%_ = [−3.74, −0.92], for disadvantageous relative to advantageous unequal splits.

When we assessed the effects of rLPFC tACS on choice behaviour, we observed that tACS_theta-sham_ significantly increased the impact of efficiency on choices depending on inequity type (for mean levels of Inequity_absolute_), tACS_theta-sham_ × Efficiency × Inequity_type_: HDI_mean_ = 1.67, HDI_95%_ = [0.22; 3.13] (Figure 2C–D, Table 2). This suggests that theta tACS over rLPFC strengthens the preference for efficient choices more strongly under disadvantageous than under advantageous inequity: When participant receive less payoff than the other, they show a stronger preference for the payoff-maximizing unequal option under rDLPFC theta tACS compared with sham. We observed no significant effects of tACS_beta-sham_ on Inequity_absolute_, Efficiency, or Inequity_type_ (Table 2), and also a further GLMM comparing theta versus beta tACS yielded no significant stimulation effects. We note that also adding a predictor for trial number (to explore for potential fatigue effects on choice behaviour) to the GLMMs for the rTPJ and rLPFC experiments revealed no significant influence of trial number on choices (HDIs_95%_ included zero), while the pattern of significant stimulation effects remained unchanged in both experiments. Taken together, our model-free results provide evidence that theta oscillations in rLPFC are causally involved in increasing the preference for options that maximize the overall welfare particularly when the decision maker is worse, rather than better, off than the other receiver. In contrast, theta tACS over the rTPJ increased inequity aversion independently of whether inequity is advantageous or disadvantageous for the decision maker.

**Table 2.** Results of Bayesian GLMM for the dictator game in the rLPFC experiment. We report the upper and lower borders of the 95% HDI of the posterior distributions. SEM are in brackets. Significant effects are highlighted in bold

Predictor	Estimate (SE)	2.5%	97.5%
**Intercept**	**6.71 (1.25)**	**4.52**	**9.40**
tACS_theta-sham_	0.36 (0.41)	−0.40	1.18
tACS_beta-sham_	−0.03 (0.42)	−0.86	0.80
**Inequity_absolute_**	**−1.57 (0.46)**	**−2.46**	**−0.64**
**Efficiency**	**4.06 (0.88)**	**2.53**	**5.97**
**Inequity_type_**	**−4.56 (1.37)**	**−7.36**	**−1.99**
Discomfort	−0.29 (0.33)	−0.92	0.37
**Inequity_absolute_** × **Inequity_type_**	**−2.21 (0.72)**	−**3.74**	−**0.92**
Inequity_absolute_ × Efficiency	−0.55 (0.52)	−1.59	0.47
**Efficiency** × **Inequity_type_**	**4.59 (1.30)**	**2.34**	**7.35**
tACS_theta-sham_ × Inequity_type_	−0.14 (0.51)	−1.14	0.86
tACS_beta-sham_ × Inequity_type_	−0.39 (0.49)	−1.35	0.61
tACS_theta-sham_ × Inequity_absolute_	0.38 (0.41)	−0.40	1.23
tACS_beta-sham_ × Inequity_absolute_	0.23 (0.38)	−0.49	0.99
tACS_theta-sham_ × Efficiency	−0.68 (0.51)	−1.70	0.30
tACS_beta-sham_ × Efficiency	−0.57 (0.49)	−1.56	0.35
tACS_theta-sham_ × Inequity_absolute_ × Inequity_type_	−0.66 (0.59)	−1.82	0.48
tACS_beta-sham_ × Inequity_absolute_ × Inequity_type_	−0.17 (0.54)	−1.23	0.86
**tACS_theta-sham_** × **Efficiency** × **Inequity_type_**	**1.67 (0.73)**	**0.22**	**3.13**
tACS_beta-sham_ × Efficiency × Inequity_type_	1.28 (0.75)	−0.15	2.90
tACS_theta-sham_ × Inequity_absolute_ × Efficiency	−0.42 (0.62)	−1.61	0.81
tACS_beta-sham_ × Inequity_absolute_ × Efficiency	0.45 (0.56)	−0.61	1.58
tACS_theta-sham_ × Inequity_absolute_ × Efficiency × Inequity_type_	1.49 (0.80)	−0.08	3.09
tACS_beta-sham_ × Inequity_absolute_ × Efficiency × Inequity_type_	0.03 (0.73)	−1.36	1.48

As robustness check, we also assessed whether simpler models (omitting interactions between Inequity_absolute_, Inequity_type_ and Efficiency) explain the data better than the full factorial model (including all interaction terms). For this purpose, we computed eight additional GLMMs for each the rTPJ and the rDLPFC experiment and compared the variance in choices explained by these GLMMs with the determination coefficient *R*^2^. In both the rTPJ and the rLPFC experiment, the full factorial model (GLM-9) explained the data better than all less complex models (Table 3; for significant results in these GLMMs, see *Supplementary* Material, Tables S3–S18).

**Table 3.** Model comparisons for the rTPJ and the rLPFC experiment. In addition to the GLMM reported in the main text, we fitted also GLMMs step-wise leaving out higher order interaction effects. We report determination coefficients *R*^2^ (as measure of explained variance) separately for the rTPJ and rLPFC experiment

GLM	Fixed effects	rTPJ	rLPFC
GLM-1	Inequity_absolute_ + Inequity_type_ + Efficiency	0.754	0.771
GLM-2	Inequity_absolute_ × Efficiency + Inequity_type_	0.762	0.781
GLM-3	Inequity_absolute_ × Inequity_type_ + Efficiency	0.763	0.774
GLM-4	Inequity_absolute_ + Inequity_type_ × Efficiency	0.757	0.774
GLM-5	Inequity_absolute_ × Inequity_type_ + Inequity_absolute_ × Efficiency	0.767	0.782
GLM-6	Inequity_absolute_ × Efficiency + Inequity_type_ × Efficiency	0.765	0.784
GLM-7	Inequity_absolute_ × Inequity_type_ + Efficiency × Inequity_type_	0.772	0.781
GLM-8	Inequity_absolute_ × Inequity_type_ + Efficiency × Inequity_type_ + Efficiency × Inequity_absolute_	0.775	0.788
GLM-9	Inequity_absolute_ × Inequity_type_ × Efficiency	0.776	0.790

In addition to these model-free GLMMs, we analysed choice behaviour in the dictator game also with computational models of inequity aversion. Model comparisons revealed that in both experiments a Fehr–Schmidt model with separate terms for advantageous and disadvantageous inequity aversion explained the data better (mean AIC_rTPJ_ = 64; mean AIC_rLPFC_ = 57) than a single-parameter model (mean AIC_rTPJ_ = 118; mean AIC_rLPFC_ = 101). When we assessed stimulation effects on parameters from the best-fitting model, theta tACS over the rTPJ non-significantly tended to increase aversion to advantageous inequity, non-parametric Wilcoxon test: *W* = 130, *P* = 0.09, without affecting disadvantageous inequity aversion, *W* = 170, *P* = 0.45. Beta rTPJ tACS showed no significant effects, all *P* > 0.13. In contrast, theta tACS over rLPFC enhanced aversion to disadvantageous inequity, *W* = 100, *P* = 0.02. We observed no further significant impact of rLPFC tACS on Fehr–Schmidt parameters, all *P* > 0.30. The results from the Fehr–Schmidt model (theta rLPFC tACS affects disadvantageous inequity aversion, theta rTPJ tACS shows a trend-level effect on advantageous inequity aversion) appear consistent with the pattern observed in the model-free analyses. We note, however, that the Fehr–Schmidt model—contrary to our model-free analysis—does not consider the influence of efficiency concerns on choices under advantageous and disadvantageous inequity, such that our model-free analyses may provide a more fine-grained picture of participants’ fairness preferences.

### Theta oscillations in rTPJ causally implement perspective taking

The observed effects of rTPJ stimulation on inequity aversion raise the question as to whether these findings can be explained by the established role of the rTPJ for perspective taking (Saxe and Kanwisher, 2003; Frith and Frith, 2006; Van Overwalle, 2009; Schurz *et al.*, 2013). Despite the evidence that the rTPJ is causally relevant for perspective taking (Santiesteban *et al.*, 2012, 2015), the underlying brain oscillations causally implementing the ability to differentiate between one’s own and others’ perspectives remain unknown. Therefore, we tested whether rTPJ theta tACS promotes perspective taking and, if so, whether the link between rTPJ theta oscillations and inequity aversion can be explained by their role for perspective taking. We hypothesized that the impact of rLPFC tACS on inequity aversion, in contrast to the rTPJ, is unrelated to perspective taking processes.

To test our hypotheses, we analysed the influences of rTPJ and rLPFC tACS on performance in the director task. In control trials, participants could stick with their own perspective to identify the object designated by the director, whereas in experimental trials participants needed to inhibit their own perspective to resolve the conflict between their own and the director’s perspective. We regressed performance in the director task (correct versus incorrect responses) on predictors for tACS_theta-sham_, tACS_beta-sham_, Condition (control = 0, experimental = 1), and the interaction terms.

**Fig. 3.**
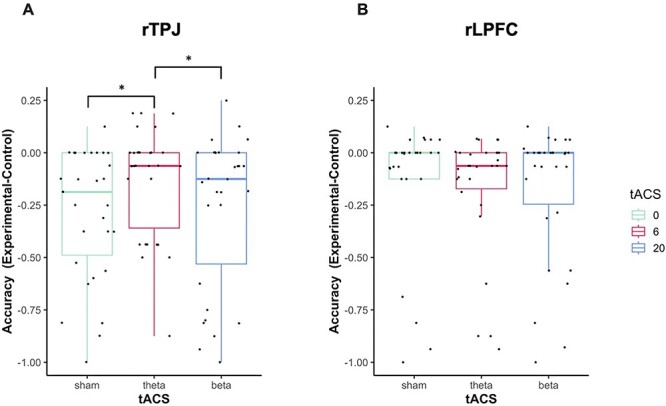
Results of tACS effects on accuracy (perspective taking) in the director task. (A) rTPJ theta tACS, compared with sham tACS and beta tACS, increased accuracy in the experimental condition relative to the control condition. (B) rLPFC tACS showed no significant effects on accuracy in the director task. Coloured boxes indicate median and interquartile range, black dots indicate individual data points (*N* = 30 participants). Values are calculated based on the difference between each participant’s accuracy rates in the experimental and the control condition: in experimental trials participant needed to switch to the directors perspective, whereas in control trials participants could stick with their own perspective. More negative values indicate worse performance in the experimental relative to the control condition, reflecting the need to resolve conflicts between one’s own and the director’s perspective.

In line with previous studies (Santiesteban *et al.*, 2012, 2015), we found a significant effect of Condition on accuracy in the rTPJ experiment, HDI_mean_ = −2.21, HDI_95%_ = [−3.37; −0.94], suggesting that participants committed more errors when their perspective was incongruent, compared to congruent, with the director’s perspective. The significant tACS_theta-sham_ × Condition interaction, HDI_mean_ = 1.56, HDI_95%_ = [0.55; 2.54], suggested that (as hypothesized) rTPJ theta tACS effects on accuracy depended on whether perspectives were congruent or incongruent, whereas we observed no significant tACS_beta-sham_ × Condition interaction, HDI_mean_ = 0.53, HDI_95%_ = [−0.50; 1.58] (Table 4). Post-hoc GLMMs showed that in the experimental condition theta tACS increased accuracy in contrast to sham, HDI_mean_ = 1.57, HDI_95%_ = [1.07; 2.14] (Figure 3), whereas we could not find significant effects of theta tACS in the control condition: tACS_theta-sham_, HDI_mean_ = 0.92, HDI_95%_ = [−0.65; 3.11]. tACS_beta-sham_ affected performance neither in experimental, HDI_mean_ = 0.25, HDI_95%_ = [−0.47; 1.10], nor in control trials, HDI_mean_ = -0.33, HDI_95%_ = [−1.36; 0.80]. Additionally, GLMMs comparing the effects of theta versus beta tACS revealed that the influence of theta tACS on performance in experimental versus control trials was significantly stronger than the influence of beta tACS, tACS_theta-beta_ × Condition, HDI_mean_ = -1.28, HDI_95%_ = [−2.28; −0.23] (Figure 3 and Table 5). Thus, rTPJ theta tACS, relative to both sham tACS and beta tACS, improved participants’ ability to inhibit their own perspective in order to resolve conflicts between their own and the director’s perspective.

**Table 4.** Results of Bayesian GLMM for the director task in the rTPJ experiment. We report the upper and lower borders of the 95% HDI of the posterior distributions. SEM are in brackets. Significant effects are highlighted in bold

Predictor	Estimate (SE)	2.5%	97.5%
**Intercept**	**3.62 (0.31)**	**3.04**	**4.31**
tACS_theta-sham_	−0.02 (0.44)	−0.85	0.90
tACS_beta-sham_	−0.33 (0.43)	−1.15	0.59
**Condition**	**−2.21 (0.63)**	−**3.37**	−**0.94**
Discomfort	−0.10 (0.20)	−0.49	0.31
**tACS_theta-sham_** × **Condition**	**1.56 (0.51)**	**0.55**	**2.54**
tACS_beta-sham_ × Condition	0.53 (0.54)	−0.50	1.58

**Table 5.** Results of Bayesian GLMM for the director task in the rTJP experiment comparing theta with beta tACS. We report the upper and lower borders of the 95% HDI of the posterior distributions. SEM are in brackets. Significant effects are highlighted in bold

Predictor	Estimate (SE)	2.5%	97.5%
**Intercept**	**3.52 (0.34)**	**2.93**	**4.25**
tACS_theta-beta_	−0.26 (0.40)	−1.02	0.55
Condition	−0.32 (0.70)	−1.69	1.11
Discomfort	0.05 (0.22)	−0.36	0.50
**tACS_theta-beta_** × **Condition**	**−1.28 (0.51)**	**−2.28**	**−0.23**

When conducting the same GLMMs for the rLPFC experiment, neither theta nor beta tACS significantly affected performance in the director task in contrast to sham tACS, tACS_theta-sham_ × Condition: HDI_mean_ = −0.03, HDI_95%_ = [−1.06; 1.00], tACS_beta-sham_ × Condition: HDI_mean_ = 0.46, HDI_95%_ = [−0.60; 1.52] (Table 6), and we also observed no significant differences between theta and beta tACS, tACS_theta-beta_ × Condition: HDI_mean_ = 0.41, HDI_95%_ = [−0.54; 1.42] (Table 7). Consequently, contrary to the rTPJ, there was no evidence for rLPFC involvement in perspective taking.

**Table 6.** Results of Bayesian GLMM for the director task in the rLPFC experiment. We report the upper and lower borders of the 95% HDI of the posterior distributions. SEM are in brackets. Significant effects are highlighted in bold

Predictor	Estimate (SE)	2.5%	97.5%
**Intercept**	**3.56 (0.35)**	**2.93**	**4.29**
tACS_theta-sham_	−0.15 (0.41)	−0.95	0.64
tACS_beta-sham_	−0.22 (0.40)	−0.99	0.54
Condition	−1.31 (0.73)	−2.71	0.11
Discomfort	−0.20 (0.20)	−0.61	0.18
tACS_theta-sham_ × Condition	−0.03 (0.51)	−1.06	1.00
tACS_beta-sham_ × Condition	0.46 (0.55)	−0.60	1.52

**Table 7.** Results of Bayesian GLMM for the director task in the rLPFC experiment comparing theta with beta tACS. We report the upper and lower borders of the 95% HDI of the posterior distributions. SEM are in brackets. Significant effects are highlighted in bold

Predictor	Estimate (SE)	2.5%	97.5%
**Intercept**	**3.31 (0.35)**	**2.68**	**4.05**
tACS_theta-beta_	0.05 (0.43)	−0.75	0.98
**Condition**	**−1.38 (0.66)**	**−2.64**	**−0.03**
Discomfort	−0.23 (0.31)	−0.91	0.34
tACS_theta-beta_ × Condition	0.41 (0.49)	−0.54	1.42

### Impact of rTPJ tACS on perspective taking mediates stimulation effects on inequity aversion

Given that theta tACS over the rTPJ enhanced both perspective taking and inequity aversion, we conducted a mediation analysis to test whether the rTPJ’s involvement in inequity aversion can statistically be explained by its more general role for perspective taking. For this purpose, we entered mean individual accuracy differences between experimental and control trials under theta versus sham tACS from the director task as additional predictors to the GLMM we had used to analyse rTPJ tACS effects on choices in the dictator game (Baron and Kenny, 1986; Preacher and Hayes, 2008). Re-computing this GLMM revealed that, contrary to the original GLMM results, the effect of tACS_theta-sham_ on inequity aversion no longer passed the statistical threshold, tACS_theta-sham_ × Inequity_absolute_: HDI_mean_ = −0,89, HDI_95%_ = [−1.95, 0.08]. Crucially, when we tested the significance of the indirect mediation path with a Sobel test (Sobel, 1982, 2008), the marginally significant result of this effect suggests that the impact of rTPJ theta tACS on inequity aversion can statistically be explained by tACS-induced changes in perspective taking, *z* = 1.92, *P* = 0.05. In contrast, we found no significant mediation effects for the influence of beta rTPJ tACS, Sobel test: *z* = 0.91, *P* = 0.36 or in the rLPFC experiment, Sobel test: *z* = 0.06, *P* = 0.95. Thus, the role of the rTPJ, though not of the rLPFC, in inequity aversion can be explained by its more general contribution to perspective taking.

## Discussion

Both rTPJ and rLPFC are thought to play central roles in social decision making, but their causal contributions to moderating aversion to advantageous versus disadvantageous inequity as well as the brain rhythms underlying these functions remained unknown so far. Here, we advance the field by determining the specific roles of neural oscillations in rTPJ and rLPFC for advantageous and disadvantageous inequity aversion. While entrainment of theta oscillations in rTPJ increased advantageous inequity aversion (though without showing stronger tACS effects on advantageous than disadvantageous inequity aversion), theta tACS over rLPFC showed dissociable effects depending on the type of inequity involved: theta tACS increased the preference for welfare-maximizing efficient choices more strongly for disadvantageous than for advantageous unequal splits. Moreover, our data suggest that rTPJ and rLPFC affect social decisions via dissociable cognitive mechanisms, as only theta stimulation of rTPJ, but not rLPFC, changed perspective taking processes, which statistically explained the rTPJ tACS effects on inequity aversion. Taken together, our study provides evidence for dissociable neuro-cognitive roles of theta oscillations in rTPJ and rLPFC for weighing inequity concerns against selfish interests, improving our understanding of the functions of these brain mechanisms in social decision making.

Although previous evidence suggested a causal role of rTPJ for prosocial giving (Soutschek *et al.*, 2016; Obeso *et al.*, 2018), these brain stimulation studies did not differentiate between different types of inequity. A neuroimaging study dissociating between advantageous and disadvantageous inequity reported that grey matter volume in the rTPJ predicted individual differences in advantageous, but not disadvantageous, inequity aversion (Morishima *et al.*, 2012). While our findings suggest that rTPJ stimulation indeed enhances advantageous inequity aversion, there was no evidence for dissociable effects on advantageous and disadvantageous inequity aversion, though we note that the lack of a significant difference must not be interpreted as evidence against inequity-specific contributions of the rTPJ to decision making (Soutschek *et al.*, 2016; Obeso *et al.*, 2018). Parameter estimates from the Fehr–Schmidt model revealed only a trend-level effect of rTPJ tACS on advantageous inequity aversion, such that also the model-based analyses do not allow drawing any conclusions regarding the specificity of the rTPJ for advantageous versus disadvantageous inequity. Nevertheless, our findings point to a function of the rTPJ for integrating own and others’ needs into the choice process, which increases the preference for equal splits in order to reduce the conflict between selfish and other-regarding interests. From a psychological perspective, this function may rely on the ability to distinguish between own and others’ mental states, which on the neural level is implemented by the rTPJ (Santiesteban *et al.*, 2012; Martin *et al.*, 2019, 2020; Zhang *et al.*, 2019). While a link between the rTPJ’s roles for perspective taking and social decision making has often been discussed in the literature (Baumgartner *et al.*, 2014; Strombach *et al.*, 2015; Soutschek *et al.*, 2016), our mediation analysis provides conclusive evidence that the rTPJ’s causal involvement in perspective taking indeed statistically explains its contribution to social decision making. Thus, rTPJ theta oscillations enable us to put ourselves into the shoes of others, which increases the sensitivity for conflicts between selfish and other-regarding interests at least in the domain of advantageous inequity (though there was no significant difference between advantageous and disadvantageous inequity).

A different result pattern emerged in the rLPFC tACS experiment, where theta entrainment in rLPFC increased the preference for efficient (i.e. welfare-maximizing) choice options more strongly for disadvantageous than for advantageous inequity. Results from the Fehr–Schmidt model (which did not include an efficiency term) suggest moreover increased aversion to disadvantageous inequity under rLPFC tACS. Combining the model-free and model-based findings, rLPFC theta oscillations may therefore play a causal role for disadvantageous inequity aversion: While the model-based results show that theta tACS increases aversion to disadvantageous inequity, the model-free results suggest that rLPFC tACS may strength disadvantageous inequity aversion particularly for less efficient offers, where the payoff for the decision maker is relatively low. For efficient offers, in contrast, the large selfish reward for the decision maker may compensate the aversion against disadvantageous splits. Thus, our findings extend previous accounts according to which the rLPFC is associated with rejection of unfair offers in the Ultimatum game (though the Ultimatum game does not allow disentangling disadvantageous inequity aversion and efficiency concerns) (Knoch *et al.*, 2006; Baumgartner *et al.*, 2011) or with the punishment of norm violations (Buckholtz *et al.*, 2008, 2015) by suggesting that the rLPFC may integrate fairness motives with efficiency concerns.

In addition to determining the roles of rTPJ and rLPFC in inequity aversion, our findings also provide insights into the brain oscillations implementing these functions. The causal involvement of theta oscillations in both rTPJ and rLPFC in social decision making is consistent with previous electrophysiological findings linking theta oscillations in these regions to perspective taking (Rodrigues *et al.*, 2015; Wang *et al.*, 2016; Gooding-Williams *et al.*, 2017; Seymour *et al.*, 2018) and cognitive control processes (van de Vijver *et al.*, 2011; Oehrn *et al.*, 2014), respectively. The rTPJ is a major network hub and implements perspective taking in communication with medial prefrontal cortex (Seymour *et al.*, 2018). As communication between distant regions is thought to rely on synchronous firing in the theta rhythm (Canolty and Knight, 2010), we speculate that specifically theta rTPJ tACS improved perspective taking by increasing the connectivity between rTPJ and other parts of the mentalizing network. Note that only in the director task we found that rTPJ theta tACS affected behaviour relative to both sham and beta tACS, whereas in the dictator game we observed no significant differences between theta and beta tACS. One possible reason for this is that also beta oscillations might play a role in social decision making (though these effects did not pass the statistical threshold in the current study). Previous findings linked rTPJ beta oscillations to individual differences in prosociality (Gianotti *et al.*, 2019), which might hint to dissociable roles of theta and beta oscillations in the rTPJ for social decisions. Thus, we are cautious with any conclusions regarding the frequency-specific of our stimulation effects on inequity aversion. As further limitation, it is worth keeping the relatively low spatial specificity of tACS (and transcranial electrical stimulation in general) in mind, allowing no inferences regarding which precise subregions in the prefrontal and the parietal cortices are responsible for the observed effects. Nevertheless, our findings provide first evidence for a causal contribution of prefrontal and parietal theta oscillations to social decision making, which highlights the importance of using tACS for studying the rhythms causally underlying the communication between neural populations.

Deficits in social decision making belong to the core symptoms of several psychiatric disorders (Chang *et al.*, 2012; King-Casas and Chiu, 2012) and previous findings suggest that these deficits in social interactions are linked with dysfunctions in the prefrontal cortex and parietal regions (Schneider *et al.*, 2013; Horat *et al.*, 2018; Bitsch *et al.*, 2019; Hu *et al.*, 2021). Studying cortical oscillatory dynamics can lead to a better understanding of the neuronal mechanisms underlying deficits in cognitive and social impairments in psychiatric disorders (Kirihara *et al.*, 2012). By providing insights into how brain rhythms in prefrontal and parietal brain regions implement social decision making, our findings contribute to improving our understanding of the neural basis of altered social behaviour in psychiatric disorders.

## Data Availability

The data that support the findings of this study as well as the R code for data analysis will be available on Open Science Framework (https://osf.io/8ybp7/?view_only=11e1775f38224deaa2311aa9f93d1f80).
